# Marination increases the bioavailability of lead in game meat shot with lead ammunition

**DOI:** 10.1017/jns.2021.15

**Published:** 2021-04-06

**Authors:** Kirsten Schulz, Franziska Brenneis, Richard Winterhalter, Markus Spolders, Hermann Fromme, Silvio Dietrich, Petra Wolf, Carl Gremse, Helmut Schafft, Robert Pieper, Monika Lahrssen-Wiederholt

**Affiliations:** 1 Department of Safety in the Food Chain, German Federal Institute for Risk Assessment, 10589 Berlin, Germany; 2 Department of Chemical Safety and Toxicology, Bavarian Health and Food Safety Authority, 80538 Munich, Germany; 3 Ludwig-Maximilians-University, Institute and Outpatient Clinic for Occupational, Social and Environmental Medicine, 80336 Munich, Germany; 4 Department of Nutritional Physiology and Animal Nutrition, University of Rostock, 18059 Rostock, Germany

**Keywords:** Venison, Roe deer, Lead acetate, Animal feeding trial, Lead bioavailability

## Abstract

As a consequence of the toxicological lead characteristics, a reduction of its exposure should consider all sources. Game meat might contain elevated levels of lead due to the use of lead ammunition. The aim of the present study was to investigate the effects of acidic marination on the bioavailability of ammunition-derived lead in game meat (Roe deer), using the growing pig as an animal model. Furthermore, the study should provide evidence that the large-area scattering of lead particles leads to noticeable differences in the individual lead intake per game meat portion. Pigs of group A (*n* 7) received lead-shot game meat, which was cooked in water. Pigs of group B (*n* 7) received lead-shot game meat, which was first marinated (wine and vinegar) and then cooked. The lead content of both game meat preparations was equal with 0⋅77–0⋅79 mg Pb/portion. Pigs of group C (*n* 4) received lead-free game meat, which was also marinated and cooked. Additionally, lead acetate was administered intravenously to group D pigs (*n* 4). Blood samples were taken on elevated time points before and after game meat intake/i.v.-application. The acidic marination increased the bioavailability of orally ingested lead, resulting in significantly higher blood lead concentrations. The bioavailability of lead was 2⋅7 % when game meat was just cooked and 15 % when the meat was marinated before. The considerable variation of the individual blood lead concentrations suggests that an inhomogeneous distribution of ammunition-derived lead particles (in terms of size and number) causes individually non-comparable lead intakes from the consumption of game meat.

## Introduction

Lead is an environmental contaminant that occurs naturally from erosion or volcanism but also derives from anthropogenic sources such as mining, smelting, lead batteries and ammunition. Chronic exposure to lead is associated with an elevated risk of cardiovascular and chronic kidney diseases in adults and of impaired neurodevelopment and subsequent cognitive and behavioural development in the foetus and young children^(1)^. Even at low levels, lead exposure is associated with intellectual deficits in children^(2)^. According to the European Food Safety Authority (EFSA), there is no evidence of a threshold for a number of critical lead-induced effects. Therefore, according to the ALARA principle, ‘As low as reasonably achievable’, any additional exposure to lead should be avoided. Today, dietary intake is the main pathway of exposure to lead in European countries. According to the EFSA, lead dietary exposure ranges from 0⋅36 to 1⋅24 μg/kg body weight (BW) per d in adults, from 0⋅21 to 0⋅94 μg/kg BW per d in infants and from 0⋅80 to 3⋅10 μg/kg BW per d in children, assuming average consumption habits^(1)^. Ammunition-derived lead is a significant cause of dietary lead exposure in groups of people who frequently consume game meat^(3)^. These are mostly hunters and their families, with consumption quantities of up to 90 portions of 200 g of game meat per year^(4)^. In indigenous people in Canada, stable lead isotope ratios in blood revealed that lead ammunition is the main reason for elevated blood lead (B-Pb) concentrations^(5)^. While pork, beef and veal are regulated under Commission Regulation (EC) No. 1881/2006 with current maximum levels of 0⋅1 mg Pb/kg meat, there are presently no regulatory standards for lead in game meat. Besides the fact whether the ammunition contains lead or not^(6)^, other factors, such as the distance from where the animal was shot or where the bullet penetrated the tissue, as well as different bullet types, influence the distribution of lead tissue contamination. Thus, even tissues far away from the wound channel may contain lead particles of different quantities and sizes^(7,8)^.

For the consumers of game meat, the rate of absorption of ingested lead depends on the consumer's age, nutritional status and other individual characteristics, as well as the physical and chemical characteristics of the lead ingested^(3)^. An older study with rats showed the highest bioavailability for lead acetate, while the bioavailability of metallic lead was only 14 %^(9)^. Therefore, under the influence of gastric acid, inert lead particles may react to soluble and more bioavailable salts. However, chemical reactions and dissolution of lead ammunition fragments in the meat might have already occurred during the storage of the animal carcass, as well as during kitchen preparation and cooking when acidic components like juice, wine or vinegar are used^(10,11)^. Soaking meat in seasoned, often acidic liquids before cooking, the so-called marination, is widely used when preparing game meat. A study published in 2011 using an *in vitro* gastrointestinal simulation technique suggested a higher bioaccessibility of lead from shot pellets in game meat cooked with vinegar (6⋅8 %) and wine (4⋅5 %) compared with uncooked meat (0⋅7 %)^(11)^. Yet, *in vivo* data on the bioavailability of ammunition-derived lead in game meat are rare^(3)^. Nevertheless, there are indications that subjects who eat game meat from animals killed with lead ammunition have higher blood lead concentrations than controls^(12,13)^.

Thus, the aim of the present study was to investigate the influence of marination and cooking on the bioavailability of ammunition-derived lead in game meat using the growing pig as an animal model. The use of pigs as a relevant human medical model is well documented. Pigs and humans share similarities in the gastrointestinal microbial diversity^(14–16)^, which allows a variety of nutritional intervention approaches. It is assumed that the absorption of lead from feed in adult pigs is 5–10 %, which corresponds to the absorption rate in adult humans. Due to the more active metabolism in young animals, the absorption rate is likely to be higher. In addition, the study should provide evidence that the large-area scattering of lead particles (due to the impact of the bullet into the wildlife body) leads to a noticeable difference in individual lead intake per game meat portion.

## Material and methods

### Game meat collection

During September 2013 and February 2014, twenty-four roe deer (*Capreolus capreolus*) were hunted with lead ammunition and thirty with lead-free ammunition in different regions of the German federal state of Brandenburg. Licensed hunters killed the game during the established hunting season and in accordance with German regulations (German Hunting Act; Bundesjagdgesetz) and best practices. The study did not involve any additional killing other than what is carried out in German forests on a regular and as regular management practices basis (population control). The deer were shot in the following hunting grounds: Stadtforst Strausberg, Maerkisch Luch and Bundesforstbetrieb Havel-Oder-Spree. The permit was granted by the German federal state of Brandenburg and the respective hunting authority. The lead ammunition consisted of an RWS Jacketed Softpoint round-nose projectile (caliber 7 × 64) or a GECO-Softpoint projectile (caliber 8 × 57 IS, 30-06 or .308). Animals were in the age group of 0–3 years. All animals were eviscerated according to the normal hunting practice. The carcasses were left hanging for 3–4 d at 7 °C and were stored at −24 °C until further processing.

Each carcass was processed at the German Federal Institute for Risk Assessment (BfR). The wound channel was removed, i.e. any tissue, that was visibly affected by the bullet or bloodshot, an additional 20 cm of visibly unaffected meat around the removed wound channel all together was defined as ‘marketable meat near the wound channel’. The following pieces of game meat were taken from each roe deer: shoulder, saddle, haunch and marketable meat near the wound channel. The game meat parts were then pooled and homogenised to determine the lead concentration. The homogenisation procedure is described in more detail in the following paragraphs.

### Animal study

#### Diet preparation

The respective edible parts (shoulder, saddle, haunch and marketable meat near the wound channel) were collected separately in order to prevent cross-contaminations and to prepare the different diets. The animal feeding trial included three different meat preparations: Two preparations were derived from lead-shot game meat, while for a third preparation, game hunted with lead-free ammunition was used. Furthermore, one part of the lead-shot game meat was just cooked and the other part was marinated for 24 h before cooking. The lead-free game meat was marinated (24 h) and cooked, too. The acidic marinade consisted of 0⋅25 litres of apple vinegar, 0⋅5 litres of red wine and 0⋅5 litres of water (pH = 4⋅15; pH meter: PCE-228M; electrode: CPC-OSH-12-01). The meat was first seared in a pan with 40 ml of vegetable oil and then cooked for 1 h/kg meat, with either one-third of water or marinade. Afterwards, the meat (including the cooking liquid) was homogenized in a commercial meat cutter (LeMaTec Voelklingen, Standkutter DMK 20 SF). Accordingly, the final homogenate consisted of two-thirds of game meat and one-third of cooking liquid. The pH values of the homogenates were recorded and a representative sample of 100 g of each homogenate was taken for lead analysis. For this purpose, four to five individual samples of 20–25 g each were taken per homogenate and mixed to form a total sample. The aim of the homogenization and the composition of the homogenate samples from several individual samples was to minimise the unknown distribution of the lead particles (in terms of quantity and size) as far as possible.

Because the game meat near the wound channel had the highest concentration of lead (Table 1), this part was used for the bioavailability study with growing pigs. The lead contents were as follows: Lead-shot game, which was just cooked: 2⋅17 mg/kg; lead-shot game, which was first marinated and then cooked: 0⋅54 mg/kg. Due to the different levels of lead in the two preparations, the portion size of the game meat had to be adjusted, subsequently. This was to ensure that all pigs, irrespective of the preparation method, ingested the same amount of lead. A maximum lead intake of 0⋅77–0⋅79 mg per portion seemed feasible. The procedure is described in detail in the following paragraphs.

**Table 1.** Concentration of lead (mg/kg) in different parts of game meat after homogenization and different preparation procedures

		Kitchen preparation^a^	
Sample side	Ammunition	Cooked	Marinated + cooked
Near wound channel^b^	Lead	2⋅17	0⋅54
	Lead-free		–
Saddle^c^	Lead	<0⋅02	<0⋅02
	Lead-free		<0⋅02
Shoulder	Lead	<0⋅02	<0⋅02
	Lead-free		–
Haunch	Lead	<0⋅02	0⋅21
	Lead-free		–

a Differentiation of the types of preparation: Lead-shot game meat were either just cooked or marinated (24 h; vinegar-wine-mixture) and cooked. Lead-free shot game meat was marinated (24 h; vinegar-wine-mixture) and cooked.

b Near wound channel: marketable meat within a radius of 20 cm around the removed wound channel.

c In the lead-free hunted game, only the saddle was examined for lead concentration.

#### *In vivo* bioavailability study and feeding diet regimen

The animal study was conducted with growing Danish Duroc boars and consisted of a feeding trial and an intravenous (i.v.) application of lead acetate. The pigs were taken from the farm Gut Schweinezucht Alt-Gaarz Bluecherhof GmbH & Co. Agriculture KG. Pigs were vaccinated against circovirus, mycoplasmas and Shiga toxin before the start of the trial. The experiments were in line with the Animal Welfare Act and licensed by the Ethics Committee of the federal state of Mecklenburg-Western Pomerania, Germany (Landesamt für Landwirtschaft, Lebensmittelsicherheit und Fischerei; LALLF 7221.3-2-027/15; 7221.3-2-021/16). The experiments were carried out at the experimental station of the University of Rostock, Faculty of Agriculture and Environmental Sciences (Department Nutritional Physiology and Animal Nutrition). The animals were housed according to their feeding groups in climate-controlled buildings on fully slatted concrete floor. Feed and water were offered *ad libitum*. All pigs were fed with a commercial dry feed for pigs (ATR Porco Baby III NDS, Werk Sollerup, Sollerupmühle, 24852 Sollerup alpha DE SH 00006). The animals were monitored several times a day for their state of health. In doing so, symptoms of a disturbed general condition were observed and noted when they occurred.

A total of 18 male pigs, about 6 weeks old, were randomly divided into three feeding groups (mean BW of 13⋅4 ± 0⋅6 kg). The pigs of feeding group A (*n* 7) received game meat that was lead-shot and cooked (game meat near the wound channel with 2⋅17 mg Pb/kg meat). The pigs of group B (*n* 7) received game meat that was lead-shot, marinated for 24 h and cooked (game meat near the wound channel with 0⋅54 mg Pb/kg meat). Due to the different lead concentrations of these homogenates (Table 1) and with the aim of achieving a similar lead intake of feeding groups A and B, the meat portion was cut in different sizes for both groups. The portion size of group A was 355 g and for group B it was 1470 g. This should ensure a uniform lead intake for groups A and B of 0⋅77 to 0⋅79 mg per serving. Pigs of feeding group C (*n* 4) received a portion of 450 g of lead-free (<0⋅02 mg/kg) game meat (saddle; marinated and cooked). With respect to the larger portion size, the total quantity of meat was offered in several smaller portions of 300–350 g in group B. When the trough was empty, the next portion was refilled. The animals of group B had one full day to eat. All pigs were housed in individual boxes exclusively for the experimental game meat feeding. Each animal had a cleaned feeding trough and a nipple drinker. All meat refusals were collected and weighed to calculate the actual lead intake per pig. The experimental game meat portion was offered to the animals once. After completion of the game meat feeding, all animals went back to their groups and received the commercial pig feed again.

During an initial adaptation period of 2 weeks, the animals of groups A, B and C were fed a lead-free game meat portion (the feeding was staggered, starting with 100–300 g for pigs of groups A and C and starting with 100–1000 g for pigs of group B) on days 9, 6 and 2 before the experimental game meat portion was fed.

To calculate the absolute bioavailability of ammunition-derived lead, i.v.-application of lead acetate (lead-(II)-acetate trihydrate solution; Carl Roth, purity: > 99⋅5 %, Karlsruhe, Germany) was applied to four pigs with a mean BW of 16⋅9 ± 0⋅4 kg (group D). For this purpose, lead-(II)-acetate was dissolved in a physiological saline solution. The presumed concentration of 0⋅263 mg Pb/ml was not confirmed by the laboratory analysis. According to the analysis, the concentration of the applied lead acetate was 0⋅133 mg/ml, which corresponds to 0⋅4 mg of lead acetate in 3 ml of saline solution. The application of 3 ml of this solution was done via the *Vena auricularis* (WDT cannula 18G × 1 1/2, 1⋅2 × 40 mm).

Seven blood samples were taken per animal of groups A, B, C and D: on time point 0 (24 h prior to game meat intake/i.v.-application) as well as 2, 24, 48, 72, 120 and 168 h after the game meat intake/i.v.-application. Blood samples (7⋅5 ml) were taken from the *Vena cava cranialis* and kept in special tubes for metal analysis (Sarstedt S-Monovette, coated with Lithium-Heparin 92 × 15 mm). Samples were stored at −21 °C until analysis.

### Lead analysis

Lead analysis in meat and blood was done by the Bavarian Health and Food Safety Authority, Department of Chemical Safety and Toxicology.

#### Lead concentration in game meat

A homogenized game meat sample of 0⋅8 g was mixed with 4 ml of nitric acid and 0⋅5 ml of hydrochloric acid. Each sample was placed in a high-pressure Teflon container for microwave pressure digestion according to EN 13805:2014. Analysis of lead was done via Inductively Coupled Plasma-Mass Spectrometry (ICP-MS; Quadrupole, Type 7700, Co. Agilent, Waldbronn, Germany).

#### Lead concentration in blood

Blood samples were at least in part coagulated. Therefore, the whole sample was mixed with two parts of concentrated nitric acid and shook for several hours at room temperature until the blood/nitric acid mixture was homogenized. From each blood sample, two probes of 3 ml were pretreated by microwave digestion using quartz vessels. Rhodium was added as an internal standard to account for losses during microwave digestion. The recovery rate of lead in the blood samples was determined several times during the period of blood sample preparation with a lyophilised whole blood control standard (ClinCheck-Control level 1, obtained from Recipe Chemicals and Instruments GmbH, Munich, Germany). The recovery rate for lead in blood was found to be 88 % (standard deviation of 8 %, *n* 18). An aliquot (1 ml) of the digested blood sample was diluted 1:10 with ultrapure water, and rhenium was added as an internal standard for ICP-MS-analysis (NexION 300D, PerkinElmer). The limit of detection was 0⋅002 μg/l for the common lead-isotope ^208^Pb and even lower for other lead isotopes.

### Statistical analysis

In the experiment, a difference in the bioavailability of at least 68 % was targeted as statistically significant, with a first type error of at most 0⋅05 and a second type error of at most 0⋅20. Using the program G*Power and the functionality ‘*t*-tests, Means: Difference between two independent means (two groups), A priori: compute required sample size - given α, power and effect size. Two tails’, it could be concluded that seven animals are needed in groups A and B^(17)^.

The pH value of the different preparation types (with or without marination) and the lead concentrations in the blood of the pigs after the game meat meal/i.v.-application of lead acetate were statistically evaluated. Statistical analysis was done using IBM SPSS Statistics 21, using the Multivariate Linear Model. The normal distribution of the data was confirmed using the Kolmogorov–Smirnov test. The significance level was *P* < 0⋅05. In addition, the lead concentrations in the blood of the individual animals were graphically displayed using Microsoft Excel to visualise the individual scattering of the concentrations within a group, to provide information on the homogeneous distribution of lead particles in the game meat. This allows conclusions to be drawn about a varying lead content of the individual game meat portions.

The area under the curve (AUC) for the B-Pb concentration was calculated using the linear trapezoidal rule^(18)^:
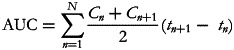


For AUC, the lead concentration in the blood on time point 0 (24 h prior to game meat intake/i.v.-application ≙ background contamination) was subtracted from the lead concentrations in the blood after the intake of game meat/i.v. application (individually for each pig). The aim was to calculate the bioavailability only by using the lead concentration that was proved to result from the consumption of the game.

The absolute bioavailability *F* for the orally ingested lead was calculated as the result of the dose-dependent AUC after oral consumption of game meat, divided by the dose-dependent AUC after the lead - i.v.-application:
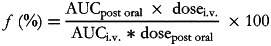


The dose after oral ingestion corresponded to the concentration of lead ingested by actual consumption of the game meat. This concentration was calculated for each animal individually using backweighing (of the game remaining in the feeding troughs).

## Results

The health status of the animals was checked daily. Negative impacts on health or behaviour were not observed during the experiment. There was no difference in daily weight gain between groups, with 710 g/d in group A, 681 g/d in group B, 708 g/d in group C and 789 g/d for pigs of the i.v.-group (*P* = 0⋅197).

### pH value of the game meat

Table 2 shows the pH values (mean ± sd) in the different game meat samples according to the different types of preparation. There was no difference in the pH value regarding the different edible parts of the game (*P* = 0⋅876) within the type of preparation. Therefore, the pH value across the different sample sides was 6⋅63 ± 0⋅06 when the game meat was just cooked and 5⋅58 ± 0⋅06 when the game meat was marinated for 24 h and cooked (*P* = 0⋅006).

**Table 2.** pH values (means ± sd) in different edible parts of lead-shot game meat depending on the type of preparation

Sample side	Preparation^a^	pH value
Near wound channel^b^	Cooked	6⋅47 ± 0⋅17
	Marinated + cooked	5⋅66 ± 0⋅17
Saddle	Cooked	6⋅59 ± 0⋅17
	Marinated + cooked	5⋅51 ± 0⋅17
Shoulder	Cooked	6⋅75 ± 0⋅17
	Marinated + cooked	5⋅70 ± 0⋅17
Haunch	Cooked	6⋅73 ± 0⋅17
	Marinated + cooked	5⋅45 ± 0⋅17
*P*-value		0⋅006

a Differentiation of the type of preparation: Lead-shot game meat was either just cooked or marinated (24 h; vinegar-wine-mixture) and cooked. Lead-free shot game meat was marinated (24 h; vinegar-wine-mixture) and cooked.

b Near the wound channel: marketable meat within a radius of 20 cm around the removed wound channel.

### Lead intake

The lead intake was calculated taking into account the game meat refusals. The animals of groups A and C consumed their game meat portion within 20 min. With the exception of one pig, the animals of group B did not consume the whole portion. Accordingly, mean Pb intakes were 0⋅77 ± 0⋅00 mg in group A and 0⋅65 ± 0⋅12 mg in group B. Within group B, the animal with the lowest intake consumed 734 g game meat (≙ 0⋅40 mg Pb), while the animal with the highest intake consumed 1⋅47 kg game meat (≙ 0⋅80 mg Pb).

### B-Pb concentrations, calculation of AUC and absolute Pb bioavailability

**Fig. 1.**
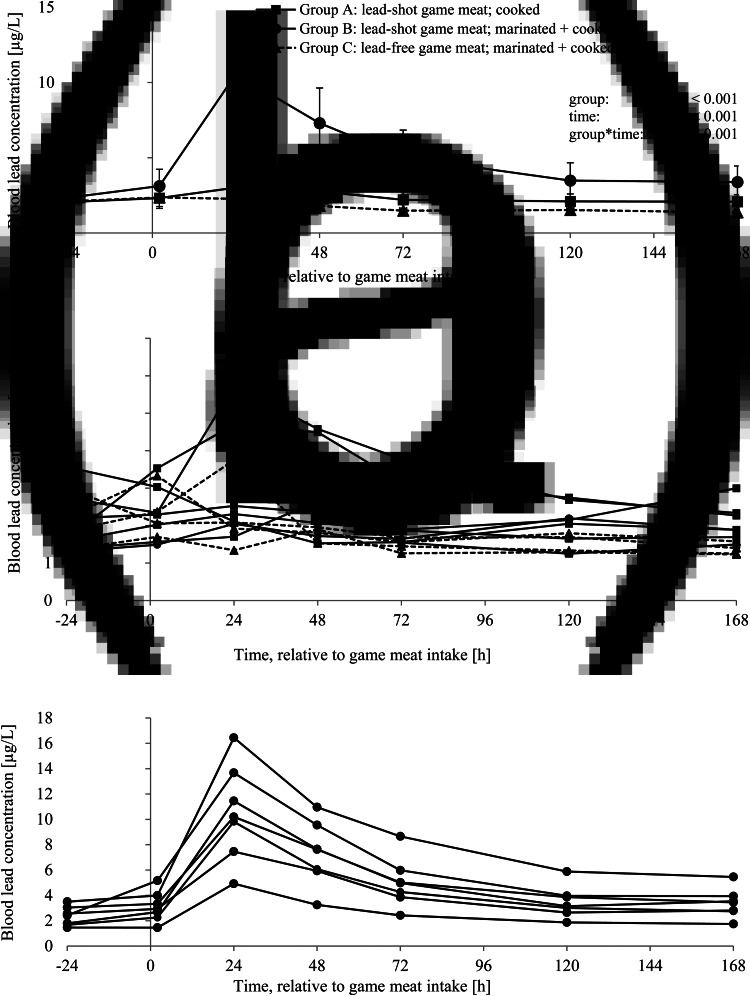
(a). Blood lead concentrations (mean ± se) in pigs following intake of differently prepared game meat (groups A, B and C). (b) Blood lead concentration in individual pigs following game meat intake of group A and group C. (c) Blood lead concentration in individual pigs following game meat intake of group B.

**Fig. 2.**
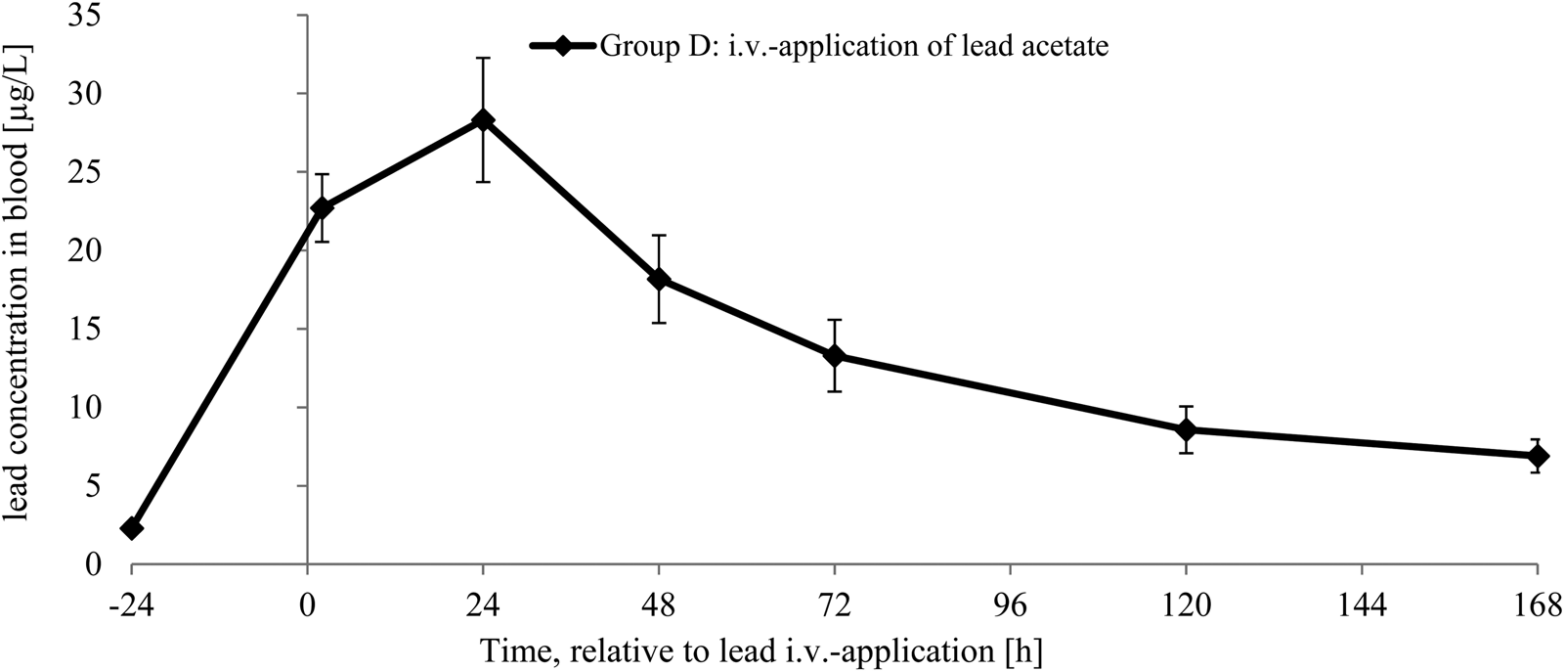
Blood lead concentration in pigs of group D (mean ± se) after intravenous application of 0⋅4 mg lead acetate via *Vena auricularis.*

Fig. 1 shows the B-Pb concentrations over time in feeding groups A, B and C (Fig. 1(a): mean ± sd) as well as B-Pb concentrations of individual pigs. The high dispersion of B-Pb concentrations within a group is visually represented in Fig. 1(b) (group A *v.* group C) and 1(c) (group B). B-Pb concentrations (mean ± sd) of group D are demonstrated in Fig. 2.

There was a significant difference in B-Pb concentrations between groups A and B (*P* < 0⋅001) and between groups B and C (*P* < 0⋅001), but not between groups A and C (*P* = 0⋅756). For groups A and B, as well as for group D, B-Pb concentration peaked 24 h after the meal/i.v.-application. The mean peak concentrations were 3⋅02 ± 1⋅44 μg/l (group A), 10⋅57 ± 3⋅53 μg/l (group B) and 28⋅31 ± 3⋅95 μg/l (group D). The mean peak concentration of group D animals is exhausted by the pig of group B with the highest B-Pb concentration (16⋅45 μg/l) to almost 60 %. The increase to peak concentration was significant (*P* < 0⋅001) for groups B and D with Δ = 8⋅22 μg/l and Δ = 26⋅02 μg/l, respectively, compared with the initial blood sample 24 h before the meal/i.v.-application. For group A, this increase was not significant (Δ = 0⋅97 μg/l, *P* = 0⋅173), and there was no increase in B-Pb in the case of group C. Seven days after ingestion of game meat, pigs of group B showed the highest B-Pb concentrations with a mean concentration of 3⋅38 μg/l (group A: 2⋅08 μg/l, group C: 1⋅37 μg/l). This corresponded to 38⋅5 % higher B-Pb levels in group B *v.* group A at the end of the trial (not significant). The individual concentration 24 h after the meal ranged from 1⋅70 to 5⋅75 μg/l (group A), from 4⋅93 to 16⋅45 μg/l (group B) and from 1⋅34 to 3⋅75 μg/l (group C). The mean background exposure (24 h prior to game meat consumption/i.v.-application) across all groups ranged between 2⋅05 and 2⋅35 μg/l.

The means of the calculated values for AUC and the resulting absolute bioavailability are shown in Table 3. Marinating game meat before cooking increased the bioavailability of lead to 15 % compared with 2⋅7 % in unmarinated meat. Hence, the mean bioavailability was about 5⋅6 times higher in orally ingested, lead-shot game meat when the meat was marinated before cooking. The absolute bioavailability of individual animals in group B varied between 9⋅2 and 23⋅4 %. For the animals in group A, the range was 0⋅5–7⋅3 %. For one animal in group A, bioavailability could not be calculated, because the lead concentrations measured in the blood were below the background concentration (24 h prior to game meat consumption).

**Table 3.** Area under the curve (AUC) values and absolute bioavailability of ammunition-derived lead after oral ingestion in pigs

Treatment^a^	Dose (mg)	AUC	Absolute bioavailability (%)
i.v. (*n* 4)	0⋅400	2057⋅4	
A (*n* 6)	0⋅774	106⋅9	2⋅7
B (*n* 7)	0⋅649	500⋅6	15⋅0

a The pigs of the i.v.-group received an intravenous application of 0⋅4 mg lead acetate via *Vena auricularis*. The pigs of group A received lead-shot game meat. The game was cooked. For one animal in group A, bioavailability could not be calculated, because the lead concentrations measured in the blood were below the background concentration (24 h prior to game meat consumption). Pigs of group B received lead-shot game meat. The game was marinated (vinegar-wine-mixture) and cooked.

## Discussion

When lead ammunition was used for hunting, elevated levels of lead were analysed in homogenized game meat, especially near the wound channel and to a lesser extent in the haunch. The levels of lead in game meat were comparable to those levels reported from a German research project on the levels of lead in roe deer. Although the mean lead contents of game meat close to the wound channel were considerably higher (4728 mg lead/kg game meat), median (0⋅025 mg/kg) and P95 (2⋅24 mg/kg) offer lower, comparable, results between both investigations^(7)^. The higher lead contents may be due to manifold factors affecting bullet fragmentation as well as the much higher number of hunted roe deer.

Furthermore, the present results show that an acidic marinade significantly increases the bioavailability of ammunition-derived lead in wild game meat. To date, little information exists on the bioavailability of ammunition-derived lead. However, possible health effects strongly depend on the bioavailability of lead from inert fragments of consumed meat. We used the growing pig as a model for humans and specifically children due to its similar anatomical and functional properties of the digestive tract^(14–16)^. In a previous study, B-Pb concentrations in pigs increased up to 23 μg/l 2 d following ingestion of lead-fragment containing game meat, while in control animals, the level was 6⋅3 μg/l^(19)^. However, the rate of bioavailability could not be calculated from this experiment, because the exact amount of lead in the meat portions was unknown. In the present study, the calculated absolute bioavailability was 15 % for the ammunition-derived lead when game meat was marinated before cooking as compared with 2⋅7 % for lead in non-marinated meat. This was close to or within the bioavailability range (3–21 %) that was previously proposed by the EFSA^(1)^. Because the bioavailability also depends on age and physiological status, Green and Pain concluded that the ratio of bioavailability of ammunition-derived lead in children is comparable to the lead bioavailability from ordinary food in adults^(20)^. Hence, when comparing the bioavailability of lead with further investigations, the age of the used animals should also be taken into account. Nevertheless, variations in the bioavailability of lead are mainly due to the different chemical binding forms of lead. For example, metallic lead is less bioavailable than, e.g., lead acetate or lead sulphide^(9)^. Lead of ammunition is an alloy, e.g. with antimony. Exposed to air, the surface of metallic lead is quickly converted to lead oxide. In turn, lead oxide dissolves in physiological fluids^(21)^. Therefore, the entry of a bullet into the animal body might further initiate changes in the chemical binding structures of lead ammunition fragments to more bioavailable compounds. It was shown that the transformation to lead acetate continues during transport and storage of the animal carcass^(10)^ and can additionally increase under the influence of acidic components during kitchen preparation (e.g. via marination of game meat)^(10,22)^. Accordingly, it seems reasonable that lead in game meat is at least partly present as lead acetate, the lead salt of the acetic acid. Hence, the acidic marination of lead-shot game meat caused an increased chemical transformation of ammunition lead (i.e. metallic lead) to lead salts like lead acetate that are more bioavailable. This resulted in higher B-Pb concentrations.

Our observation is in agreement with a Spanish investigation that evaluated the transfer of lead from shot to meat during the preparation of breasts of quails. The birds were not shot but spiked with a various number of lead-shot fragments. It was shown that the use of lead shot for small game hunting contributes significantly to the contamination of this meat, with higher levels of lead in the meat when it was cooked with vinegar instead of water^(22)^. In the present study, in which we used rifle ammunition instead of lead shot, peak B-Pb concentration in pigs was reached 24 h after game meat ingestion and was 3⋅5 times higher for the pigs of group B compared with the pigs of group A, even though the mean intake of orally ingested lead was smaller in group B (Δ = 0⋅125 mg Pb/portion). Furthermore, the B-Pb concentration in group B pigs still showed an increase by 1⋅03 μg/l at the end of the trial compared with the initial blood sample (negative control). Therefore, our results support the hypothesis that acidic ingredients used during the preparation of game meat increase the bioavailability of ammunition-derived lead, which was demonstrated by analysing B-Pb concentrations within an animal feeding trial. Furthermore, the present results indicate that individual B-Pb concentrations vary strongly. The visual representation of the individual animal graphs shown in Fig. 1 was chosen particularly because there was no significant difference in the mean B-Pb concentrations of groups A and C. However, the single-animal representation shows that the consumption of lead-shot game meat, which was not marinated, also leads to individual increases in the B-Pb concentration (Fig. 1(b)). This may be relevant when considering the health risk of lead. Plotting the data reveals the single peak concentrations of lead of up to 5⋅74 μg/l in group A and an individual bioavailability of up to 7⋅3 % could be calculated, even though the mean lead intake was obviously comparable between pigs of group A as all animals feed the whole game meat portion. At least two (out of seven, about 28 %) group A pigs show a sharp increase in the B-Pb concentration after consumption of the game meat portion, although the meat for group A was not marinated and no acidic digestion was provoked by kitchen preparation. As an example from group B, the animal with the third-highest game meat intake had the highest B-Pb concentrations (peak concentration: 16⋅45 μg/l; Fig. 1(c)), corresponding to an absolute bioavailability for the ammunition-derived lead of 23 %, which is almost 10 % above the average. Within group B, pigs were fed different amounts of game meat. This might affect the individual scattering of the lead concentrations in the blood of the single animals. For the calculation of the bioavailability, however, the apparently real amount of lead ingested per animal was determined (by backweighing the game meat residuals in the feeding trough). Since the absolute bioavailability also varies between 9⋅2 and 23⋅4 % within group B, the different game meat intakes cannot be the sole cause of the variations. Possible reasons for this variation may, of course, be physiological, since bioavailability is subject to biological variation. However, the main reason might be that the game meat portions contained lead fragments of different sizes, even in micro- and nanogram quantities. Smaller lead particles are more likely to dissolve completely due to their larger surface-to-volume ratio compared with larger particles. Kollander *et al.* detected lead particles <40 nm in game meat samples more than 10 cm away from the wound channel^(10)^. Fragments like these remain in the game body even after the removal of the wound channel. As detected particle number concentration can range from 27 to 50 million particles/g meat and lead particles of up to 750 nm are detectable via ICP-MS^(10)^, a homogenous distribution of the lead fragments in the single game meat portions of our investigation could not be guaranteed. In summary, it can be assumed that the lead content of the individual portions was highly variable and the individual game meat portions actually had higher or lower lead concentrations than the defined 0⋅77 to 0⋅79 mg lead per serving. Furthermore, it can be assumed that the individual game meat portions contained lead fragments that were bioavailable to different extents in terms of size and surface condition. Possibly, an improved distribution of lead fragment residues can be achieved by the use of computer tomographic measurements with device-typical detection limits of 500 μm^(23)^ or via spiking the meat with a known concentration of lead particles in a defined size. Hunt *et al.* pursued a similar scientific approach, as the presence of lead fragments was verified by X-ray technology^(19)^. Further research on this is presently being carried out by the German Federal Institute for Risk Assessment^(24)^. Blood lead concentrations are also related to deficiencies of essential nutrient elements such as iron and calcium. For example, both lead and calcium have a high affinity to the same intracellular receptors and carrier proteins. Therefore, high calcium concentrations are able to reduce the bioavailability of lead, as there are less binding possibilities for lead. As a first barrier, the intestinal mucosa counteracts the lead intake due to its regulatory function regarding calcium homeostasis. It appears that the positive influence of calcium on lead reduction is based on the physiological calcium state and to a lesser extent on the intestinal calcium concentration^(25)^. In the present investigation, the calcium and iron status of the animals was not checked. It was assumed that the supply of calcium and iron met the animal's individual requirements.

In conclusion, the results show that residues of ammunition lead are bioavailable and the bioavailability is increased by the influence of acidic marination. However, statistical analysis and a comparison of the mean values do not provide any information on the extent to which individual particle scatter (including different particle sizes) influences the bioavailability of ammunition lead. Considering the individual animals, it can be assumed that lead particles in different numbers and sizes were ingested by each animal. A homogeneous distribution of lead particles in game meat was apparently not achieved in the present experiment. Further investigations should focus on the use of spiked game meat. In any case, the use of lead-free hunting ammunition has to be preferred in the light of the possible health impact for groups exposed to this toxic dietary contaminant.
